# Primary care diagnostic and treatment pathways in Dutch women with urinary incontinence

**DOI:** 10.1080/02813432.2022.2036497

**Published:** 2022-02-18

**Authors:** Miranda C. Schreuder, Nadine A. M. van Merode, Antal P. Oldenhof, Feikje Groenhof, Marlous F. Kortekaas, Hedy Maagdenberg, Johannes C. van der Wouden, Henk van der Worp, Marco H. Blanker

**Affiliations:** a Department of General Practice and Elderly Care medicine, University Medical Center Groningen, University of Groningen, Groningen, The Netherlands; b Julius Center for Health Sciences and Primary Care, University Medical Center Utrecht, Utrecht University, Utrecht, The Netherlands; c National Health Care Institute, Diemen, The Netherlands; d Department of General Practice, Amsterdam University Medical Center, Vrije Universiteit, Amsterdam Public Health Research Institute Amsterdam, The Netherlands

**Keywords:** General practice, guideline adherence, primary health care, treatment, urinary incontinence, women

## Abstract

**Objective:**

To investigate how GPs manage women with urinary incontinence (UI) in the Netherlands and to assess whether this is in line with the relevant Dutch GP guideline. Because UI has been an underreported and undertreated problem for decades despite appropriate guidelines being created for general practitioners (GPs).

**Design:**

Retrospective cohort study.

**Setting:**

Routine primary care data for 2017 in the Netherlands.

**Subjects:**

We included the primary care records of women aged 18–75 years with at least one contact registered for UI, and then extracted information about baseline characteristics, diagnosis, treatment, and referral to pelvic physiotherapy or secondary care.

**Results:**

In total, 374 records were included for women aged 50.3 ± 15.1 years. GPs diagnosed 31.0%, 15.2%, and 15.0% women with stress, urgency, or mixed UI, respectively; no diagnosis of type was recorded in 40.4% of women. Urinalysis was the most frequently used diagnostic test (42.5%). Education was the most common treatment, offered by 17.9% of GPs; however, no treatment or referral was reported in 15.8% of cases. As many as 28.7% and 21.7% of women were referred to pelvic physiotherapy and secondary care, respectively.

**Conclusion:**

Female UI is most probably not managed in line with the relevant Dutch GP guideline. It is also notable that Dutch GPs often fail to report the type of UI, to use available diagnostic approaches, and to provide appropriate education. Moreover, GPs referred to specialists too often, especially for the management of urgency UI.

**Key points**
Urinary incontinence (UI) has been an underreported and undertreated problem for decades. Despite various guidelines, UI often lies outside the GPs comfort zone.
•According to this study: general practitioners do not treat urinary incontinence according to guidelines.
•The type of incontinence is frequently not reported and diagnostic approaches are not fully used.
•We believe that increased awareness will help improve treatment and avoidable suffering.

## Introduction

Guidelines for female urinary incontinence (UI) describe generally consistent diagnostic pathways and treatment options [1–3]. The guideline of the Dutch College of General Practitioners (GPs), for example, requires a proper patient history and voiding diary to determine the type and severity of UI [4], followed by general, abdominal, and pelvic examinations. Treatment options then differ by the type of UI, but in all cases, comorbidities should be managed properly.

Two decades ago, routine primary care data in the Netherlands indicated that UI was undertreated, with 71% of new cases not receiving active treatment, such as medication, bladder training or pelvic floor muscle training (PFMT), pessary or second-line treatment, within one year of diagnosis [5]. It has been proposed that this may result from apathy, time constraints, or lack of knowledge, with GPs often left unconfident about UI [6]. In a more recent postal survey, Dutch GPs, cited a lack of staff (43%) or time (39%) as the main reasons for non-adherence to the guideline [7]. This must also be considered in the context that patients often fail to present, further contributing the likelihood of inadequate care [8]. We wondered if the quality of care for women with UI has changed over time to meet the standard set by the Dutch GP guideline. The objective of this study was to assess the current level of care for women with UI provided by Dutch GPs, based on routine care data. We focussed on the basic assessment of patients (i.e. history taking and physical examination), additional diagnostics, and treatment including referrals.

## Material and methods

### Database

We performed a retrospective cohort study of anonymised data from a national collaboration of routine primary care data networks: Amsterdam University Medical Center – location VUmc (205,488 patients), University Medical Center Utrecht (324,374 patients), and University Medical Center Groningen (UMCG) (194,981 patients). These networks prospectively collect routine care data in a dynamic cohort. In the Netherlands, all inhabitants are registered with a single general practice, but patients may change practices, e.g. due to moving to another region, death, or other reasons. Newly registered patients will enter the cohort from the date of registration. For all patients in the cohort, data are available for many years and the electronic patient records comprised the following: pseudonymised contact data (consultations, visits, telephone, and other) recorded as free text in SOAP (Subjective, Objective, Assessment, Plan) notation; ICPC (International Classification of Primary Care) codes [9,10]; drug prescriptions based on ATC (Anatomical Therapeutic Chemical Classification System) codes; and referrals to physiotherapists and medical specialists.

### Selection of patient records

As content of care was inventoried and the study was purely descriptive, we did not perform a sample size calculation. Instead, we assumed that including 450 records of patients with UI (150 from each database) would provide a sample size that allows to describe patterns and is large enough to capture the heterogeneity in these patterns. For this, a broad automated search was done for records of women aged 18–75 years who had ≥1 contact in which a relevant ICPC or ATC code was recorded in 2017 (January 1 to December 31). Possible relevant ICPC codes were U01 (painful micturition), U02 (frequent micturition/urge), U04.01 (stress incontinence), U04.02 (urge incontinence), U04.03 (mixed incontinence), U05.01 (oliguria/anuria), U05.02 (urine retention), and U07 (other symptoms/urine complaints). The only relevant ATC code was G04BD (urological spasmolytics, including anticholinergics and mirabegron). We excluded women who had a similar contact in the 3 years before the search date (i.e. so that the cohort reflected women with new onset UI) and who had motor or visual disorders that could induce functional UI (e.g. blindness or paraplegia). Finally, the free texts of a random selection of patient records were screened by hand to confirm that UI-related care was recorded. If it was clear from the records that the consultation was about urinary tract infection, the case was ignored. We then included the confirmed UI-related cases and performed a detailed data extraction until one year after the index consultation.

We assumed beforehand that half of the screened records would qualify for data retrieval. During assessment of the first database (Groningen), however, this only applied to 3 out of 16 records. Therefore, we increased the random selection to 800 records for Groningen. Due to the ICPC/ATC coding systems of the databases at Utrecht and Amsterdam, which used less specific codes for UI, we included 1,000 records from each of those databases and applied the same screening and selection procedure (total sample, 2,800). One author per database assessed cases for selection.

### Data retrieval and outcomes

The Strengthening the Reporting of Observational Studies in Epidemiology (STROBE) Statement was applied [11]. A structured electronic case report form was used to extract the following data: GP code; birth year; UI type, severity, and duration; and pregnancy within 6 months before consulting. UI was classified as stress UI (SUI), urgency UI (UUI), mixed UI (MUI), or not specified. These were based on GP diagnosis or recorded symptoms, such as ‘incontinence due to increasing pressure’ for SUI and ‘having a sudden, intense urge to urinate followed by an involuntary loss of urine’ for UUI. We also extracted diagnostic, treatment, and referral information, including to pelvic physiotherapy and secondary care. Treatment education included records of oral explanation and/or information provision (folders or website).

As primary outcome we considered how often the type of UI was specified and how frequent diagnostic and treatment pathways and referral to second-line were initiated. For the secondary outcomes we sub-specified how frequent a specific diagnostic tool, treatment option and referral were mentioned for each type of UI.

### Analyses

The incidence of UI was estimated based on data from the three databases and the population recorded in CBS StatLine [12]. All data is reported as proportions with 95% confidence intervals where appropriate. We refrained from performing statistical analysis, other than providing descriptive statistics. Diagnostic and treatment data are presented separately for each type of UI, consistent with the Dutch GP guideline. Additionally, we calculated how many patients were prescribed incontinence pads with no further efforts at diagnostics or treatment, how many received both PFMT and physiotherapy referral, how many received both bladder training and a drug prescription, and how many were referred to gynaecology or urology with no other treatment from the GP. The anticholinergics prescribed were also recorded. All analyses were performed using IBM SPSS version 25 (IBM Corp., Armonk, NY, USA).

### Ethical considerations

The Medical Ethics Review Committee of University Medical Centre Groningen confirmed that the Medical Research Involving Human Subjects Act (WMO), which includes the Declaration of Helsinki, did not apply to our study. Screening and data retrieval occurred at the location of each database. Patients who objected to their data being used for scientific or quality of care purposes were excluded from the databases. All privacy-sensitive data were pseudonymised by a trusted third party, whereby information by which an individual can be identified has been replaced or removed. Furthermore contact data recorded as free text were anonymised before extraction.

## Results

### Incidence, type, severity, and duration of UI

**Figure 1.**
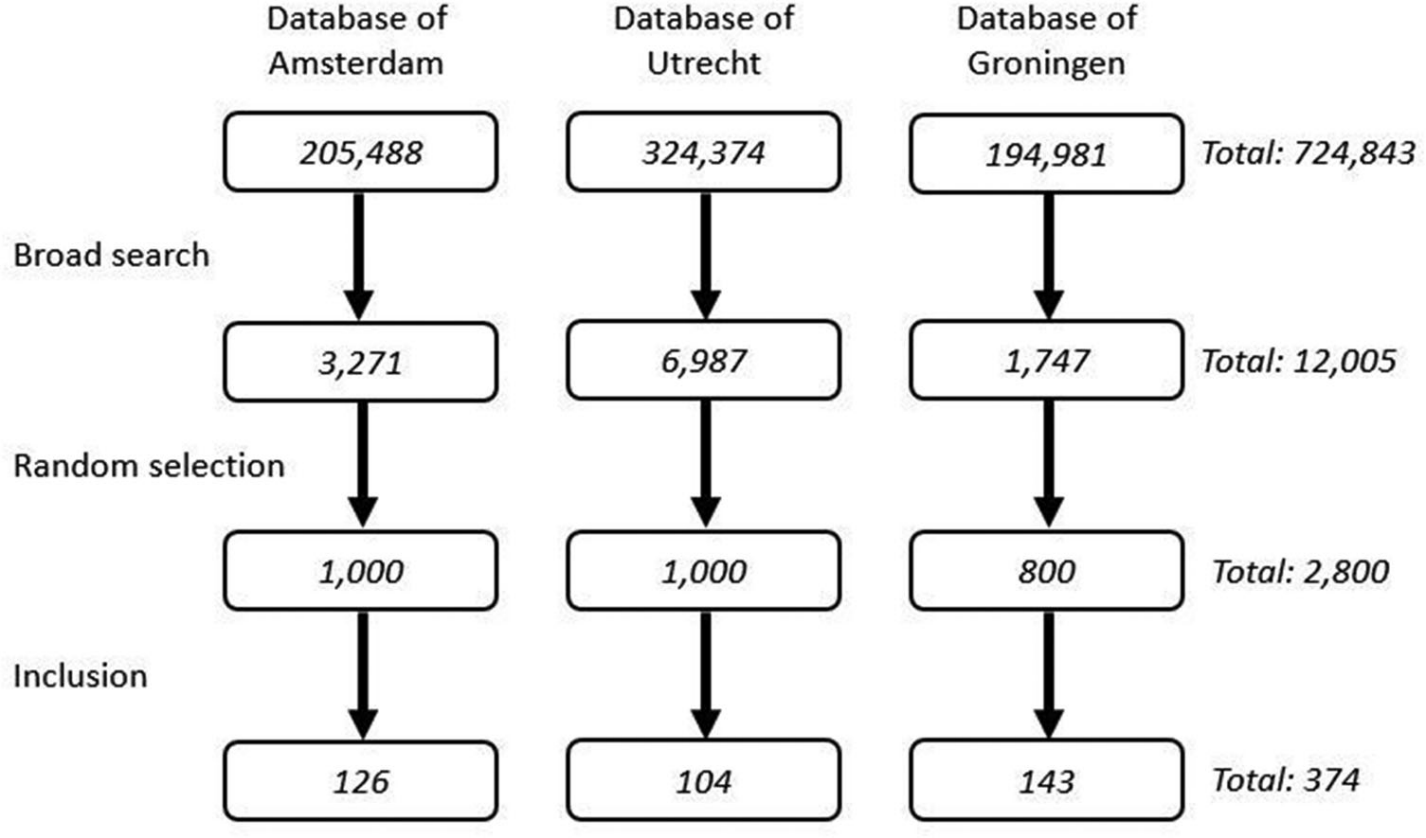
Patient selection. Of the total 724,843 records, the first search yielded 12,005 records. Screening a random selection of 2,800 records resulted in 374 women being included who received care from 183 different GPs (range, 1–9 patients per GP).

We included 374 women who received care from 183 GPs (range, 1–9 women per GP; Figure 1) and estimated an incidence of 61 per 10,000 women aged 18–75 years in the Dutch GP population (Additional file 1). As primary outcome, GPs categorised 116 (31.0%), 57 (15.2%), and 56 (15.0%) women as having SUI, UUI, or MUI, respectively. However, the type of UI was not specified in 145 cases (38.8%), or it was listed as overactive bladder/bladder spasms (*n* = 5), nocturnal enuresis (*n* = 3), or drug-induced UI (*n* = 1; olanzapine). The descriptive data are otherwise summarised in Table 1, showing a mean overall age of 50.3 ± 15.1 years, and mean ages of 46.1, 51.8, and 55.0 years for those with SUI, UUI, and MUI, respectively. Of note, UI severity was reported in only 81 (21.7%) cases and duration was reported in 131 (35.0%) cases. Only 13 (3.6%) women were pregnant in the 6 months before the index consultation (SUI = 4; not categorised = 9).

**Table 1.** Descriptive characteristics of 374 women with urinary incontinence selected from medical records.

	SUI *n* = 116	UUI *n* = 57	MUI *n* = 56	O/N *n* = 145	All *n* = 374
Age in years (Mean ± SD)	46.1 ± 13.0	51.8 ± 16.4	55.0 ± 12.1	51.3 ± 16.5	50.3 ± 15.1
Age groups (*n* (%))					
18–25	4 (3.4)	5 (8.8)	1 (1.8)	12 (8.3)	22 (5.9)
26–35	23 (19.8)	6 (10.5)	2 (3.6)	23 (15.9)	54 (14.4)
36–45	36 (31.0)	5 (8.8)	7 (12.5)	17 (11.7)	65 (17.4)
46–55	28 (24.1)	14 (24.6)	19 (33.9)	24 (16.6)	85 (22.7)
56–65	12 (10.3)	12 (21.1)	15 (26.8)	33 (22.8)	72 (19.3)
66–75	13 (11.2)	15 (26.3)	12 (21.4)	36 (24.8)	76 (20.3)
Degree of incontinence (n (%))					
≤1 episode per week	2 (1.7)	2 (3.5)	1 (1.8)	2 (1.4)	7 (1.9)
Two or three times a week	14 (12.1)	3 (5.3)	2 (3.6)	2 (1.4)	21 (5.6)
About once a day	6 (5.2)	7 (12.3)	6 (10.7)	7 (4.8)	26 (7.0)
Several times a day	4 (3.4)	6 (10.5)	8 (14.3)	5 (3.4)	23 (6.1)
All the time	0 (0.0)	1 (1.8)	2 (3.6)	1 (0.7)	4 (1.1)
Not reported	90 (77.6)	38 (66.7)	37 (66.1)	28 (88.3)	293 (78.3)
Duration of incontinence (*n* (%))					
<1 month	5 (4.3)	4 (7.0)	2 (3.6)	17 (11.7)	28 (7.5)
1 month to 1 year	7 (6.0)	9 (15.8)	3 (5.4)	10 (6.9)	29 (7.8)
>1 year	24 (20.7)	12 (21.1)	17 (30.4)	21 (14.5)	74 (19.8)
Not reported	80 (69.0)	32 (56.1)	34 (60.7)	97 (66.9)	243 (65.0)

Note 1: SUI: Stress urinary incontinence; UUI: urgency urinary incontinence; MUI: mixed urinary incontinence; O/N: Other/not reported.

### Diagnostics

As primary outcome, no diagnostics were recorded in 40.4% (95% CI: 35.5–45.3) of all cases, and this was more common for SUI and MUI than for UUI (Table 2). As secondary outcomes urinalysis was used in 159 (42.5%) cases, but with a distinct difference between UI types (63.2% for UUI; 31.2% for SUI; 30.4% for MUI). Only 88 (23.5%) cases reported external vaginal inspection and 85 (22.7%) cases reported per vaginal examination, with no differences by UI type. Pelvic floor muscle testing (9.4%) and voiding diaries (8.6%) were reported least frequently. Other diagnostic methods were ultrasound (*n* = 7), general blood tests (*n* = 7), and pap smear (*n* = 2).

**Table 2.** Diagnostics by type of urinary incontinence.

	SUI *n* = 116	UUI *n* = 57	MUI *n* = 56	O/N *n* = 145	All patients *n* = 374
Voiding diary	10.3 (4.8–15.8)	14.0 (5.0–23.0)	8.9 (1.5–16.3)	4.8 (1.3–8.3)	8.6 (5.9–11.3)
Urine tests	30.2 (21.8–38.6)	63.2 (50.6–75.8)	30.4 (18.2–42.6)	49.0 (40.8–57.2)	42.5 (37.5–47.5)
External vaginal inspection	29.3 (21.1–37.5)	22.8 (11.8–33.8)	28.6 (16.6–40.6)	17.2 (11.1–23.3)	23.5 (19.2–27.8)
Per vaginal examination	25.0 (17.2–32.8)	29.8 (17.8–41.8)	21.4 (10.6–32.2)	18.6 (12.3–24.9)	22.7 (18.4–27.0)
PFM testing	12.9 (6.8–19.0)	3.5 (0.0–8.4)	7.1 (0.2–14.0)	9.7 (4.8–14.6)	9.4 (6.5–12.3)
Other diagnostics	0.9 (0.0–2.7)	5.3 (0.0–11.2)	1.8 (0.0–5.3)	4.8 (1.3–8.3)	3.2 (1.4–5.0)
No diagnostics	50.0 (40.8–59.2)	28.1 (16.3–39.9)	42.9 (29.8–56.0)	36.6 (28.8–44.4)	40.4 (35.5–45.3)

Note 1: SUI: Stress urinary incontinence; UUI: urgency urinary incontinence; MUI: mixed urinary incontinence; O/N: Other/not reported; PFM: Pelvic floor muscle.

Note 2: Values are presented as percentage (95% CI).

Note 3: Other diagnostics consisted of ultrasound (*n* = 7), general blood test (*n* = 3) and pap smear (*n* = 2).

### Primary care treatment and referral

The treatment details are compared in Table 3, with none reported in 15.8% (95% CI: 11.1–19.5) of all cases as primary outcome. As secondary outcomes, the most frequently reported treatments included education (17.9%), incontinence pads (13.4%), and PFMT (12.6%). Overall, 106 (28.7%) women were referred for physiotherapy, with more referrals for SUI and MUI than for UUI. In 11 of the 106 (10.4%) cases referred for physiotherapy, the GP also provided PFMT. In 26 (7.0%) cases, the GP prescribed an anticholinergic (*n* = 21), mirabegron (*n* = 2), or both (*n* = 3). Anticholinergics included solifenacin (*n* = 12), tolterodine (*n* = 10), and oxybutynin (*n* = 1), with the type of anticholinergic not reported in one case. Only one case received bladder training with drug treatment. Half of the anticholinergic prescriptions were evaluated in follow-up appointments, in contrast to all mirabegron prescriptions. A total of 50 women received a prescription for incontinence pads, with 35 (70.0%) undergoing no diagnostic assessment, 34 (68.0%) receiving no other treatment, and 30 (60.0%) neither undergoing diagnostics nor receiving treatment. Finally, 81 (21.7%) cases were referred to gynaecology or urology, with slightly more referrals for SUI and UUI than for MUI; 47 of the 81 cases (58.0%) were referred without treatment.

**Table 3.** Treatment by type of urinary incontinence.

	SUI *n* = 116	UUI *n* = 57	MUI *n* = 56	O/N *n* = 145	All patients *n* = 374
**Basic treatment**					
Education during consult	19.8 (12.5–27.1)	21.1 (10.4–31.8)	28.6 (16.7–40.5)	11.0 (5.9–16.1)	17.9 (14.0–21.8)
Normal fluid intake	3.4 (0.1–6.7)	12.3 (3.7–20.9)	1.8 (0.0–5.3)	7.6 (3.3–11.9)	6.1 (3.7–8.5)
Weight reduction	0.0	1.8 (0.0–5.3)	0.0	0.7 (0.0–2.1)	0.5 (0.0–1.3)
Treating comorbidities	0.9 (0.0–2.7)	1.8 (0.0–5.3)	0.0	2.1 (0.0–4.5)	1.3 (0.1–2.5)
PFM training by GP	21.6 (14.2–29.0)	5.3 (0.0–11.2)	16.1 (6.3–25.9)	6.9 (2.8–11.0)	12.6 (9.3–15.9)
Bladder training by GP	0.0	7.0 (0.3–13.7)	7.1 (0.2–14.0)	2.1 (0.0–4.5)	2.9 (1.1–4.7)
Incontinence pads	7.8 (2.9–12.7)	8.8 (1.4–16.2)	26.8 (15.0–38.6)	14.5 (8.8–20.2)	13.4 (9.9–16.9)
Pessary	2.6 (0.0–5.5)	0.0	3.6 (0.0–8.5)	1.4 (0.0–3.4)	1.9 (0.5–3.3)
**Medication**					
Adjust medication	0.0	0.0	0.0	0.7 (0.0–2.1)	0.3 (0.0–0.9)
Start anticholinergics	0.9 (0.0–2.7)	15.8 (6.2–25.4)	5.4 (0.0–11.3)	7.6 (3.3–11.9)	6.4 (3.9–8.9)
Start mirabegron	0.0 (0.0–0.0)	1.8 (0.0–5.3)	1.8 (0.0–5.3)	2.1 (0.0–4.5)	1.3 (0.1–2.5)
Start antibiotics	4.3 (0.6–8.0)	8.8 (1.4–16.2)	7.1 (0.2–14.0)	15.2 (9.3–21.1)	9.6 (6.7–12.5)
**Other treatment**	4.3 (0.6–8.0)	8.8 (1.4–16.2)	1.8 (0.0–6.7)	3.4 (0.0–4.8)	4.3 (2.3–6.3)
**Referral**					
Pelvic physiotherapy	33.3 (24.7–41.9)	27.3 (15.3–39.3)	41.1 (28.2–54.0)	20.8 (14.1–27.5)	28.7 (24.0–33.4)
Gynaecology/urology	25.0 (17.1–32.9)	28.1 (16.3–39.9)	21.4 (10.6–32.2)	16.6 (10.5–22.7)	21.7 (17.6–25.8)
**No treatment or referral**	12.9 (6.8–19.0)	15.8 (6.2–25.4)	7.1 (0.3–13.9)	21.4 (14.7–28.1)	15.8 (11.1–19.5)

Note 1: SUI: Stress urinary incontinence; UUI: urgency urinary incontinence; MUI: mixed urinary incontinence; O/N: Other/not reported.

Note 2: Values are presented as percentage (95% CI).

Note 3: Other treatment consists of other medication (*n* = 6), relaxation exercises (*n* = 2), stop drinking caffeine (*n* = 2), using an app for treatment of incontinence (*n* = 2), Botox injection (*n* = 1), cranberry advice (*n* = 1), tampon use during exercise (*n* = 1).

## Discussion

### Statement of principal findings

The routine care data included in this retrospective cohort study suggest that the assessment and treatment of female UI in the Netherlands is probably not consistent with the Dutch GP guideline. This is accordance with a previous routine primary care data study in the Netherlands two decades ago [5]. Information on type, severity, and duration of UI was lacking in many patient files, and fewer than half of the GPs used any diagnostics. Furthermore, the included GPs tended to provide minimal education or active treatment themselves, while referring excessive numbers of women.

### Strength and limitations

We used three large databases from different regions in the Netherlands to ensure that our results offer a good representation of adherence to the Dutch GP guideline. However, electronic medical records are meant to support daily practice rather than research, and as such, may not provide all relevant patient information. Underreporting could result from GPs efficiently summarising histories and treatment plans [13] or from having a lack of awareness of UI management [14]. To reduce work duplication, we expect that GPs will report any additional diagnostics they perform, but we must emphasise that a lack of reporting data does not necessarily mean that guidelines were not followed.

### Findings in relation to other studies

Correct diagnosis is essential for appropriate treatment; however, the primary outcome type of diagnosis was not reported in 38.8% of cases, and in many cases, neither the severity nor the duration was reported. It is unknown if this represents a failure to assess or a lack of knowledge, especially given that a cross-sectional survey of Canadian GPs reported that only 35% felt comfortable dealing with UI [15]. Consistent with a previous Danish and German study showing that the consultation rate increases with frequency, severity, and duration of UI [16], and with the known barriers to seeking help [17], most women in our cohort had UI for at least 1 year before visiting a GP.

Worldwide, 50%, 14%, and 32% of women are estimated to have SUI, UUI, and MUI, respectively [18]. Although this correlates well with our rate for SUI, our rates MUI were much lower. This could be explained by our population age as the prevalence of MUI increases in women over 80 years [19]. Another possible explanation is that GPs might have included women with MUI in the category with the predominant symptoms (either urgency or stress incontinence) or in the not-specified category. Moreover, the mean annual incidence of UI is estimated at 1–9% [20], which is higher than our estimate of 0.61%. However, the Dutch Institute for Health Research (NIVEL) has estimated a prevalence of 0.6% based on routine primary care data over 1 year [21].

The Dutch GP guideline recommends urinalysis to exclude urinary tract infection because this can mimic the symptoms of UUI [4], and consistent with this, our GPs performed urinalysis in 63.2% of women with UUI. Nevertheless, patients may have been asked to take a urine sample before their consultation to allow for routine diagnostics, irrespective of the UI type, potentially explaining the high overall percentage undergoing urinalysis.

We expected physical examination rates to be higher because this is an easy and quick tool in a first consultation. However, an audit of implementing UK guidelines revealed that GPs felt incompetent doing a complete gynaecological exam, and the authors concluded that awareness should be increased during GP training [14]. Furthermore, encounters in which multiple problems are raised could lead to a lack of time for physical examination, with only the newest or most important problems likely to be recorded by GPs [13,14].

Voiding diaries are indispensable when assessing UI in most women, helping to detail behaviour and voiding frequency [22,23]. According to the Dutch GP guideline, voiding diaries can give more insight, especially if the medical history is not clear enough on the severity and frequency of incontinence. Furthermore, voiding diaries are also a useful tool to evaluate the effect of treatment. Consistent with the low percentages of completed voiding diaries seen in a previous Dutch study [7], we could not assess this in our data. Compliance issues also affect diary use because they can be seen as a burden for many patients [24]. This is especially problematic for 7-day diaries, which has resulted in 3-day voiding diaries being recommended in the Dutch GP guideline [4]. GPs may also feel that completing a voiding diary will not affect their decision. Further research is needed on this topic.

Women with UI have indicated that lack of knowledge is a barrier to seeking help [17], with education during consultations deemed critical. Although we found that GPs often failed to record education in the patient’s file, this may have been because they saw it as obvious.

The Dutch GP guideline recommends that incontinence pads only be offered as an interim to definitive treatment or as an adjunct to ongoing therapy, being reserved for long-term use only after all treatment options have been explored [1,4]. We anticipated that incontinence pad use would be higher in our study given that a study of 314 women in Dutch primary care revealed that 87% used some type of incontinence pad [25]. However, that study did not differentiate between prescribed and freely bought incontinence pads. It is also possible that GPs did not consider incontinence pads as a supportive option for use alongside other treatments, with more than half of the women prescribed pads undergoing no diagnostics or receiving no other treatment. This represents a missed opportunity that deprives women of potentially curative therapy.

Bladder training is the mainstay of treatment for UUI according to Dutch GP guidelines [4]. Despite the plethora of evidence on their benefits [26], GPs rarely prescribed anticholinergic drugs. They should be considered if bladder training is ineffective. In line with a report by the Dutch Institute of Responsible Medication Use [27], we found that solifenacin and tolterodine were the preferred drugs. However, this conflicts with the Dutch GP guideline that recommends tolterodine with extended-release or transdermal oxybutynin, based on lower costs and comparable safety and effectiveness, as side effects are common [4]. The guidelines also recommend that treatment should be evaluated after 4 to 6 weeks [1,4].

The Dutch GP guideline details clear criteria for referral to specialist care: SUI not benefiting from PFMT, severe SUI where the patient wants surgical management, or UUI not benefiting from bladder training or medication. Given that surgical management is a proven good therapeutic option for severe SUI, referral can be an appropriate first step [28]. However, bladder training can be highly effective for UUI [29], and as such, the high referral rate without first attempting bladder training reflects inadequate treatment by GPs in this cohort. By contrast, and following the guidelines, no woman with SUI received bladder training.

## Conclusion and implication

Although Dutch GPs do not appear to manage UI in line with established guidance, we believe that the problem must be tackled from both patient and GP perspectives. The current Dutch GP guideline for UI is currently not used to its full extent, but we believe that re-evaluation of the guideline at this stage is not useful. Understanding why the guideline is not optimally used is the first step in further research. A focus group study of Dutch GPs on urinary incontinence in the elderly revealed three main themes of attitudes: therapeutic nihilism of GPs, lack of time and complexity of the problem and co-morbidity [6]. We believe that GP vocational training must place greater emphasis on establishing the diagnosis and management of UI. In general, from a patient perspective, we believe in the importance of improving awareness and education, even though further research is still needed to explore this effect. Improved awareness and education could, for example, be achieved through public campaigns about potentially curative treatment options or through eHealth, which has shown promise as an emerging clinical resource. In line with this, our UMCG group is developing a mobile application that has shown promise with the main types of UI [30]. If these approaches can be combined effectively, we could truly improve the treatment of women with UI.

## Supplementary Material

Supplemental Material
