# Supplementary material for: Primary care diagnostic and treatment pathways in Dutch women with urinary incontinence
Source: Scand J Prim Health Care. 2022 Feb 18;40(1):87–94. doi: 10.1080/02813432.2022.2036497 (PMC9090412; doi:10.1080/02813432.2022.2036497)
Supplement: Supplemental Material [file IPRI_A_2036497_SM0810.docx]

**Supplementary box 1. Calculation incidence of UI in the Dutch GP population**

| **UI included in study** | 374 of 2,800 |
| --- | --- |
| **UI in broad search** | 1,603.525 of 12,005 (374 × 12,005/2,800) |
| **UI in databases** | 1,603.525 of 724,843 |
| **Women aged 18–75 in population** | 6,159,576 of 17,081,507* |
| **Women aged 18–75 in database** | 261,378 of 724,843 (6,159,576 × 724,843/17,081,507) |
| **UI incidence database** | 61 per 10,000 women aged 18–75 (1,603,525 × 10,000/261,378) |

Abbreviations: GP = general practitioner, UI, urinary incontinence.

* Centraal Bureau for Statistics. Bevolking; geslacht, leeftijd en burgerlijke staat [Dataset]. 2019; Available at: <https://opendata.cbs.nl/statline/#/CBS/nl/dataset/7461BEV/table?fromstatweb%20,%20visited%20at%2027-1-2020>. Accessed 01/27, 2020.
